# Correction to: A qualitative system dynamics model for efects of workplace violence and clinician burnout on agitation management in the emergency department

**DOI:** 10.1186/s12913-022-07560-y

**Published:** 2022-02-14

**Authors:** Ambrose H. Wong, Nasim S. Sabounchi, Hannah R. Roncallo, Jessica M. Ray, Rebekah Heckmann

**Affiliations:** 1 Department of Emergency Medicine, Yale School of Medicine, 464 Congress Ave Suite 260, New Haven, CT 06519 USA; 2 Department of Health Policy and Management, Center for Systems and Community Design, CUNY Graduate School of Public Health & Health Policy, 55 W. 125th Street, 7th Floor, New York, NY 10027 USA; 3 Department of Emergency Services, Yale New-Haven Hospital, 20 York Street, New Haven, CT 06510 USA

**Correction to: BMC Health Serv Res 22, 75 (2022)**

**https://doi.org/10.1186/s12913-022-07472-x**

Following publication of the original article [1], the authors identified that two characters are missing in the word “Mutual” of Fig. 4e’s description. “Mutu” should be changed to “Mutual”. The correct Fig. 4 is shown below.

**Figure 1:**
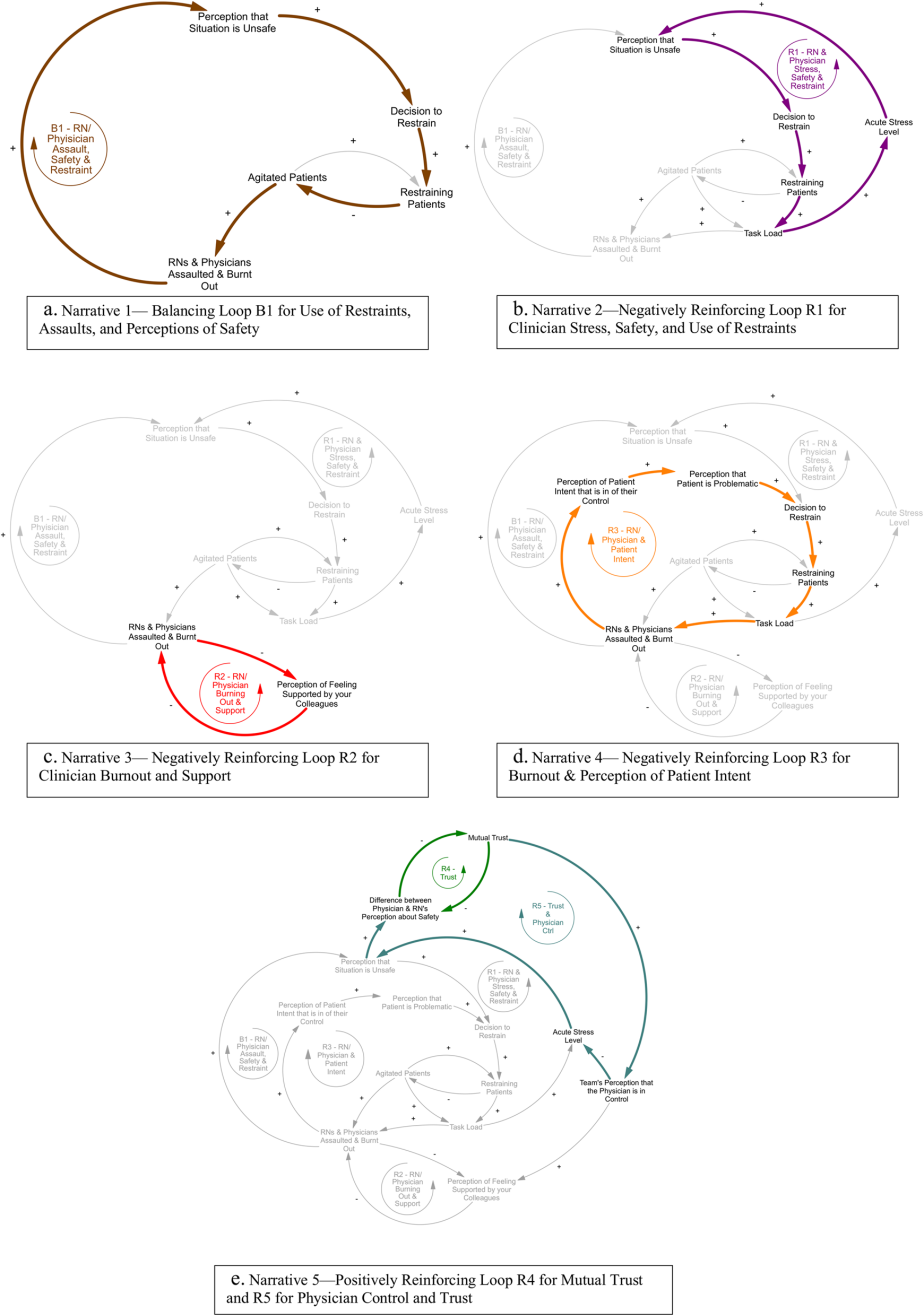
Fig. 4

In addition, an additional funder needs to be added in the Funding section, and the complete Funding section should be “Ambrose H Wong is supported by the Robert E. Leet and Clara Guthrie Patterson Trust Mentored Research Award, KL2 TR001862 from the National Center for Advancing Translational Science (NCATS), components of the National Institutes of Health and the National Institutes of Health Roadmap for Medical Research, and K23 MH126366 from the National Institute of Mental Health. The funders had no role in the design and conduct of the study; collection, management, analysis, and interpretation of the data; preparation, review, or approval of the manuscript; and decision to submit the manuscript for publication.”

The original article has been corrected.
