# Supplementary material for: A qualitative system dynamics model for effects of workplace violence and clinician burnout on agitation management in the emergency department
Source: BMC Health Serv Res. 2022 Jan 15;22:75. doi: 10.1186/s12913-022-07472-x (PMC8760708; doi:10.1186/s12913-022-07472-x)
Supplement: Supplementary file 1 — Additional file 1. [file 12913_2022_7472_MOESM1_ESM.docx]

**
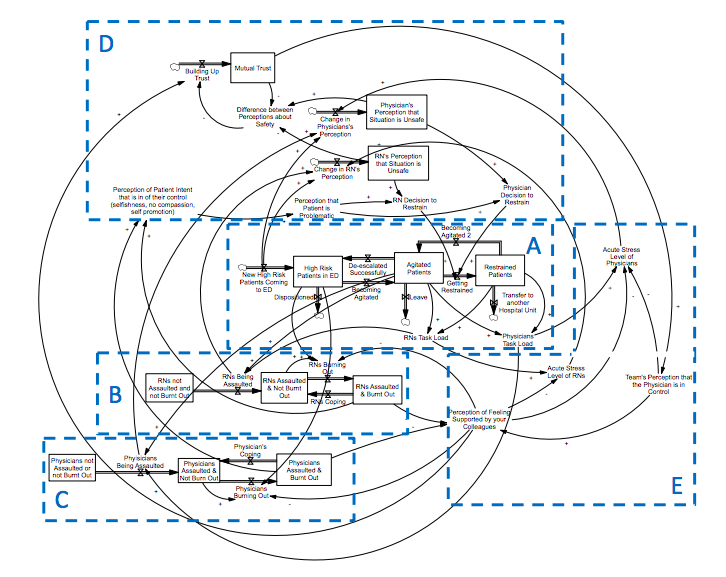
**

**Appendix 1** – Detailed Qualitative System Dynamics Model for Agitation Management, Clinician Burnout, and Decisions for Physical Restraint Use. Identified sub-sections A) Patient Flow; B) RN (Registered Nurse) Flow; C) Physician Flow; D) Perceptions of Safety and Development of Trust, and E) Perceptions of Control.

**
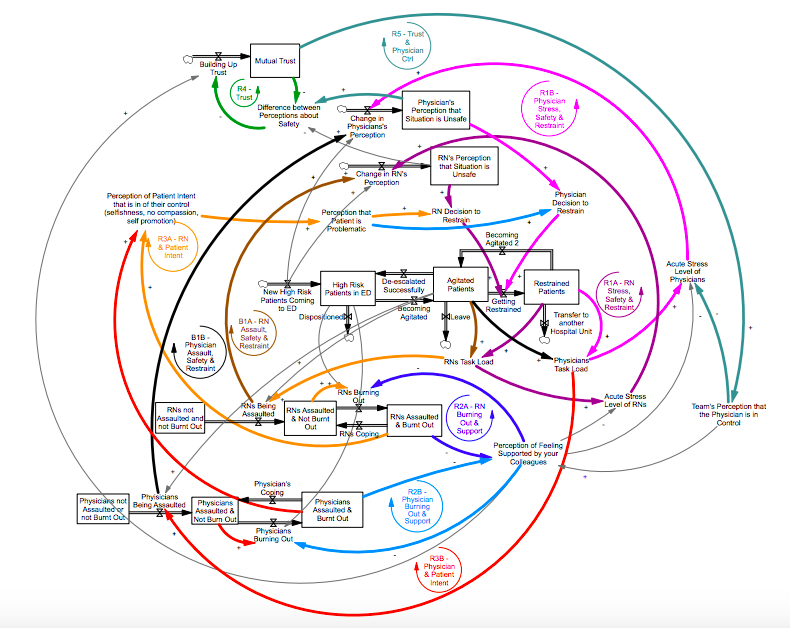
**

**Appendix 2** – Highlighted Balancing and Reinforcing Loops within detailed qualitative model. The bolded black and brown loops (B1A/B1B) represent a pair of mirrored **balancing** loops, and other complementary colors represent four pairs of mirrored **reinforcing** loops. See Appendix 3 for details of each sets of loops.

3d.

3c.

3b.

3a.

3e.

**Appendix 3** – Individual Narratives within Detailed Model.

3a. Narrative 1—Use of Restraints, Assaults, and Perceptions of Safety

(Balancing Loops B1A for Nurses and B2B for Physicians)

3b. Narrative 2—Clinician Stress, Safety, and Use of Restraints

(Negatively Reinforcing Loops R1A for Nurses and R1B for Physicians)

3c. Narrative 3—Clinician Burnout and Support

(Negatively Reinforcing Loops R2A for Nurses and R2B for Physicians)

3d. Narrative 4—Burnout & Perception of Patient Intent

(Negatively Reinforcing Loops R3A for Nurses and R3B for Physicians)

3e. Narrative 5—Development of Trust and Control

(Positively Reinforcing Loops R4 for Mutual Trust and R5 for Physician Control and Trust)
